# Fetid Sweating of the Feet

**Published:** 1888-08

**Authors:** 


FETID SWEATING OF THE FEET.—The *Kriegs-Sanitas Ordnung* recommends a powder composed of three parts of salicylic acid, ten of starch, and eighty-seven of talc. Five grammes suffice for one application, but this remedy being not always convenient, the Prussian military laws prescribe a salicylic suet (salicylic acid, two parts; mutton suet, 100 parts).—*Archives de Med. et de Pharm. Militaires.*

